# Home hospitalization for palliative cancer care: factors associated with unplanned hospital admissions and death in hospital

**DOI:** 10.1186/s12904-021-00720-7

**Published:** 2021-01-26

**Authors:** Vincent Gamblin, Chloé Prod’homme, Adrien Lecoeuvre, André -Michel Bimbai, Joël Luu, Pierre-Alexandre Hazard, Arlette Da Silva, Stéphanie Villet, Marie-Cécile Le Deley, Nicolas Penel

**Affiliations:** 1 Palliative Care Unit, Oscar Lambret Center, 3 rue Frédéric Combemale, 59300 Lille, France; 2 Palliative Care Unit, Lille University Hospital and Medical School, 59000 Lille, France; 3 ETHICS (Experiment, Transhumanism, Human Interactions, Care and Society) – EA7446, Lille Catholic University, 59800 Lille, France; 4 Direction of Research and Innovation, Oscar Lambret Center, 3 rue Frédéric Combemale, 59020 Lille, France; 5 58 bis rue de Vaufoynard, Rochecorbon, 37210 France; 6 Paris-Saclay University, Paris-Sud University, UVSQ, CESP, INSERM, Gif-sur-Yvette, France; 7 Lille University Hospital and Medical School, 59045 Lille, France

**Keywords:** Palliative care, Home hospitalization, Hospital readmission, End‐of‐life care

## Abstract

**Background:**

Home hospitalization at the end of life can sometimes be perturbed by unplanned hospital admissions (UHAs, defined as any admission that is not part of a preplanned care procedure), which increase the likelihood of death in hospital. The objectives were to describe the occurrence and causes of UHAs in cancer patients receiving end-of-life care at home, and to identify factors associated with UHAs and death in hospital.

**Methods:**

A retrospective, single-center study (performed at a regional cancer center in the city of Lille, northern France) of advanced cancer patients discharged to home hospitalization between January 2014 and December 2017. We estimated the incidence of UHA over time using Kaplan-Meier method and Kalbfleish and Prentice method. We investigated factors associated with the risk UHA in cause-specific Cox models. We evaluated factors associated with death in hospital in logistic regressions.

**Results:**

One hundred and forty-two patients were included in the study. Eighty-two patients (57.7 %) experienced one or more UHAs, a high proportion of which occurred within 1 month after discharge to home. Most UHAs were related to physical symptoms and were initiated by the patient’s family physician. A post-discharge palliative care consultation was associated with a significantly lower incidence of UHAs. Sixty-five patients (47.8 % of the deaths) died in hospital. In a multivariate analysis, living alone and the presence of one or more children at home were associated with death in hospital.

**Conclusions:**

More than 40 % of cancer patients receiving end of life home hospitalization were not readmitted to hospital, reflecting the effectiveness of this type of palliative care setting. However, over half of the UHAs were due to an acute intercurrent event. Our results suggest that more efforts should be focused on anticipating these events at home – primarily via better upstream coordination between hospital physicians and family physicians.

## Background

The development of palliative medicine at home was one of the main objectives of France’s 2015–2018 National Palliative Care Plan [1]. The introduction of this healthcare policy has had a significant impact on the development of palliative care activities; in its 2016 annual survey, the French National Homecare Federation reported a 8.4 % year-on-year increase in the number of patients benefitting from end-of-life home hospitalization [2]. Cancer is the most prevalent disease managed at home, and accounts for around a third of all patients. Between 2008 and 2014, end-of-life home hospitalization increased by 61 % in France, and accounted for 25 % of patients cared for at home in 2016 [3].

In France, most non-accidental deaths occur in hospital - between 58 % and 60 %, according to various studies performed between 2011 and 2016. [4–7]. Most end-of-life patients have expressed a preference to die at home [8–12]. Of the 333,291 adult patients who died in France in 2016, 18,664 (5.6 %) died during homecare, and 16,611 of the latter (89 %) had received palliative care [13].

Home hospitalization is aimed at patients living at home requiring complex care with significant technical expertise (complete toilet, infusions, analgesia by PCA < pump (Patient-Controlled Analgesia), enteral / parenteral nutrition, complex dressings, etc.). In general, this type of care requires 2 to 3 nursing visits per day, providing healthcare services over the full 24 hour day, 7 days a week, if needed.

Despite these measures, end-of-life home hospitalization can be perturbed by an unscheduled hospital admission (UHA, defined as any hospital admission that is not part of a preplanned care procedure), which increases the likelihood of death in hospital [14]. In the United States and United Kingdom, an excessively high frequency of UHAs even led in the past to financial penalties [15, 16].

Unscheduled hospital admissions and deaths in hospital can sometimes be seen as a failure to stay at home, if the wish of the patient (and his family) was to die at home. Indeed, patients at the end of their life who expressed their wishes for the place of death mostly favor a death at home [8–12]. Keeping patients in palliative care at home therefore appears to be an objective of quality of care.

The primary aim of this study was to describe the incidence of UHAs (at least one readmission, cumulative readmission rate during the first 12 months after the first discharge from hospital) and causes of UHAs in cancer patients receiving palliative care at home by a home hospitalization provider. We also investigated the factors associated with UHAs and death in hospital.

## Methods

### Study design

We retrospectively studied cancer patients discharged from the Oscar Lambret Center (a regional cancer center in Lille, northern France) to palliative home care.

Data were collected from patient’s hospital records, and patients were not contacted directly. Consequently, approval by an institutional review board was not required. The study complies with the MR004 reference methodology adopted by the French Data Protection Authority (Paris, France), and we checked that patients did not object to the use of their data for research purposes.

### Eligibility criteria

The main eligibility criteria were age 18 or over, a diagnosis of cancer, and the provision of palliative care by a home hospitalization provider (Santelys, Lille, France, who regularly works in coordination with the Oscar Lambret Center) after discharge from the Oscar Lambret Cancer Center between January 2014 and December 2017.

### Study objectives and endpoints

To describe the cumulative incidence of UHA, we considered the time interval from the date of hospital discharge to the date of the first UHA; observations were censored at the date of last follow-up for patients still alive at home without UHA, and death without UHA was considered as a competing event. We also extracted the causes of first UHA (acute intercurrent or new event, uncontrolled or refractory symptoms, intervention technical expertise, decline of general condition, caregiver burnout, …) and the origin of the request (family physician, hospital staff, nurse, patient’s family, home hospitalization coordinating physician) from the patient file.

The definitions of acute intercurrent event and *uncontrolled or refractoy symptoms* are :

- Acute intercurrent event : any acute pathology, which may be due to a sudden decompensation of an underlying medical condition, or an unexpected occurrence of an acute medical condition.
- Uncontrolled or refractory symptoms : main symptoms of discomfort already present at the time of initial hospitalization, and which showed a gradual worsening, rapid or not, requiring the patient’s unplanned hospitalization.

To describe the multiple readmission rate, we considered the cumulative number of UHAs from initial hospital discharge until death with the dates of successive UHA. We also computed the total duration of home hospitalization by summing the durations of the successive home hospitalizations if any.

Secondary endpoints also included the place of death (in hospital or not) and the overall survival duration, defined as the time interval between discharge from the hospital and patient death, regardless of the cause of death.

### Statistical analysis

Continuous variables were described as the mean (standard deviation) or the median (range), and categorical variables were presented as the number (percentage).

To estimate the occurrence of UHA over time after hospital discharge (median time to UHA, probability of UHA within 1 month), we used two statistical methods. The Kalbfleish and Prentice method provides an estimate of the cumulative incidence from hospital discharge where we consider death without UHA as a competing event. This estimate reflects the observed probability of UHA over time from hospital discharge in the study population; it cannot tend towards 100 % as part of the patients die without prior UHA. We also used the Kaplan-Meier method where we classify death without UHA as a censoring event. With this second method, the probability of UHA over time is computed conditionally upon being alive. We studied factors associated with the risk of UHA over time in cause-specific Cox regression models, with death without UHA as a censoring event. Cause-specific hazard ratios of UHA (cs-HR) were estimated with their 95 %-confidence intervals (95 %-CI). Prognostic value of factors associated with a *p*-value < 0.20 in univariate analyses was then evaluated in a multivariate cause-specific Cox model.

We estimated the cumulative number of UHAs during the first 12 months after the first discharge from hospital by considering successive readmissions for the same patient over time, using Nelson Mean Cumulative Function [17]. We illustrated the individual trajectories and repeated UHAs for each patient from initial hospital discharge to death or last follow-up, using a swimmer plot.

The overall survival curve was estimated using the Kaplan-Meier method.

To determine factors associated with death in hospital, we performed logistic regression models and estimated odds ratio (OR) with their 95 % confidence intervals, first in univariate analysis, then in multivariate analysis considering all factors associated with a p-value < 0.20 in univariate analyses.

The threshold for statistical significance was set to *p* < 0.05.

All statistical analyses were performed using Stata software (version 15.0, StataCorp LLC, College Station, TX).

## Results

### Study population

A total of 152 patients were screened for inclusion in the study. Ten were then excluded because of home hospitalization for postoperative care (*n* = 5), lack of data on the hospital stay (*n* = 4), and age under 18 (*n* = 1). Hence, 142 patients (88 women and 54 men) were included. The median age was 62 years (range, 26–89). One hundred and twenty-two patients (88.4 %) lived in their own home, 14 lived with relatives (10.1 %), and only one lived in a nursing home (Table 1). Before hospital discharge, the median length of hospital stay was 11 days (range, 0–79).

**Table 1:** – Patient characteristics at the time of initial hospital discharge (n = 142)

Parameters	Categories	n	%
Sex	Men	54	38.0
Age	< 50 [50–65] > 65	27 60 55	19.0 42.3 38.7
Karnofsky Index at the time of initial hospital discharge	10%20%30%40%50%60%	1186041175	0.712.742.328.912.03.5
Primary tumor site (MD = 3)	Breast Head and neck Digestive Gynecological Lung Urological Sarcoma / other	41 28 21 16 14 11 8	28.9 19.7 14.8 11.3 9.9 7.7 5.6
Current home (MD = 4)	Patient’s own home Relatives’ home Nursing home/other	122 14 2	88.4 10.1 1.5
Marital status (MD = 8)	Married Divorced Widow(er) Single Living together	85 16 14 11 8	63.4 11.9 10.4 8.2 6.0
Patient living alone (MD = 1)	Yes	28	19.9
Caregiver at home (MD = 21)	Yes	103	85.1
One or more children at home (MD = 2)	Yes	25	17.9
Initial discharge from	Medical oncology department Hospital palliative care unit	88 54	62.0 38.0
Length of hospital stay before discharge	< 8 days [8-21] > 21	46 68 28	32.4 47.9 19.7
Assessment by the mobile hospital’s palliative care team before discharge (MD = 54)	Yes	38	43.2
Psycho-oncology consultation (MD = 1)	Yes	38	27.0
Written advance directives	Yes	7	4.9
Pre-emptive prescription of sedation	Yes	24	16.9

*MD* number of missing data

*Only patients hospitalized in the medical oncology department could be assessed by the mobile palliative care team

### First UHA

Overall, 82 of the 142 patients (57.7 %) were re-admitted to hospital at least once. There were 135 UHAs in total. Fifty-eight (40.8 %) of the 142 patients died at home without being readmitted to hospital. In two cases, home hospitalization was discontinued without readmission to hospital because end-of-life care was managed by the family physician and not by the home hospitalization provider.

The median time to the first UHA conditional upon to be alive was 23 days (95 %-CI: 15–34; range: 1–164) according to the Kaplan-Meier method, and 42 days using the Kalbfleisch and Prentice method with death without UHA as a competing risk. The probability of a UHA within 1 month of discharge to home was 57 % according to the Kaplan-Meier method, and 44 % according to the Kalbfleisch and Prentice method.

The reason for the first UHA was known in 80/82 cases, as follows: an acute intercurrent event (*n* = 45, 56.3 %), refractory or uncontrolled symptoms (*n* = 9, 11.3 %), care requiring technical expertise (*n* = 9, 11.3 %), deterioration of the patient’s general condition (*n* = 5, 6.3 %), caregiver burnout (*n* = 5, 6.3 %), and other causes (*n* = 7, 8.7 %). The UHA was primarily initiated by a family physician (in 35.6 % of the first UHAs and 33 % of the subsequent UHAs) (Table 2).

**Table 2:** – Description of the first UHA

Variables	Categories	n	%
Number of UHAs	0 1 >=2	60 54 28	42.3 38.0 19.7
Admission to (MD = 1)	Palliative care unit Emergency room Medical oncology department Another unit	33 31 15 2	40.7 38.3 18.5 2.5
Admission requested by (MD = 23)	A family physician Hospital staff A nurse The patient’s family A home hospitalization coordinating physician	21 18 13 5 2	35.6 30.5 22.0 8.5 3.4
Causes (MD = 2)	Acute intercurrent or new event: o *Cardiovascular event* o *Digestive event* o *Pain* o *Hemorrhage* o *Infection* o *Delirium* o *Dyspnea* o *Iatrogenic event* Uncontrolled or refractory symptoms: o *Pain* o *Dyspnea* Intervention requiring technical expertise: o *Blood transfusion* o *Other* Decline in general condition Caregiver burnout Other causes	45 *1* *8* *3* *1* *10* *7* *13* *2* 9 *5* *4* 9 *2* *7* 5 5 7	56.3 11.3 11.3 6.3 6.3 8.7
Outcome of hospital stay after the UHA	Home hospitalization Death Transfer to another hospital	40 40 2	48.8 48.8 2.4

*MD* number of missing data

We found three factors significantly associated with UHA in multivariate analysis. Patients discharged from the palliative care unit had a higher risk than those discharged from the medical oncology department (cs-HR = 1.99; 95 %-CI, 1.21–3.27; *p* = 0.006). A higher frequency of visits by a family physician was also significantly associated with UHA (cs-HR = 1.37; 95 %CI, 1.24–1.52; *p* < 0.001) whereas patients who had a post-discharge palliative care consultation had a lower risk of UHA than those who did not (cs-HR = 0.35; 95 %CI, 0.16–0.75; *p* = 0.007). None of the other characteristics available upon hospital discharge was found statistically associated with UHAs (Table 3).

**Table 3:** – Factors associated with UHAs

Parameters	Categories	n	Univariate analysis of the risk of UHA		Multivariate analysis of the risk of UHA^(1)^	
			Crude cs-HR (95 %-CI)	p	Adjusted cs-HR (95 %-CI)	p
At the time of initial hospital discharge						
Sex	Women Men	88 54	1 1.28 (0.82–2.03)	0.28	-	
Age (/10 years)		142	0.98 (0.83–1.18)	0.90	-	
Karnofsky index	≤ 30 % > 30 %	79 63	1 0.97 (0.63–1.51)	0.91	-	
Primary tumor site	Breast Head and neck Digestive Gynecological Lung Urological Sarcoma / other	41 28 21 16 14 11 8	1 1.24 (0.60–2.34) 2.70 (1.33–5.49) 1.61 (0.78–3.33) 1.35 (0.55–3.35) 1.78 (0.71–4.45) 1.88 (0.71–4.98)	0.20	-	
Home	Patient’s home Other	122 16	1.25 (0.54–2.88) 1	0.61	-	
Marital status	Single Married/Living together	41 93	1 0.91 (0.56–1.45)	0.68	-	
Patient living alone	No Yes	113 28	1 1.30 (0.78–2.19)	0.32	-	
Caregiver at home	No Yes	18 103	1 1.11 (0.58–2.13)	0.75	-	
One or more children at home	No Yes	115 25	1 0.93 (0.54–1.61)	0.79	-	
Initial discharge from	Medical oncology dept. Palliative care unit	88 54	1 1.35 (0.86–2.12)	0.19	1 1.99 (1.21–3.27)	0.006
Length of hospital stay before discharge	Per 10-day increment	142	1.09 (0.93–1.27)	0.30	-	
Psycho-oncology consultation	No Yes	103 38	1 0.85 (0.53–1.37)	0.51	-	
Written advance directives	No Yes	135 7	1 0.93 (0.34–2.55)	0.89	-	
Preemptive prescription of end-life sedation	No Yes	118 24	1 0.82 (0.42–1.60)	0.57	-	
After initial hospital discharge						
Palliative care consultation	No Yes	124 15	1 0.46 (0.23–0.92)	0.03	1 0.35 (0.16–0.75)	0.007
Frequency of visits by a family physician	1 per 10-day increment	142	1.36 (1.23–1.50)	< 0.001	1.37 (1.24–1.52)	< 0.001

*Cs-HR* cause-specific Hazard Ratio, estimated in Cox models, where death without UHA is classified as a censoring event

95 %-CI: 95 % confidence interval

Multivariate model includes the three factors: Initial discharge from medical oncology department versus palliative care unit, Palliative care consultation, and Frequency of visits by a family physician after initial hospital discharge

### Multiple UHAs and total duration of home hospitalization

**Fig. 1:**
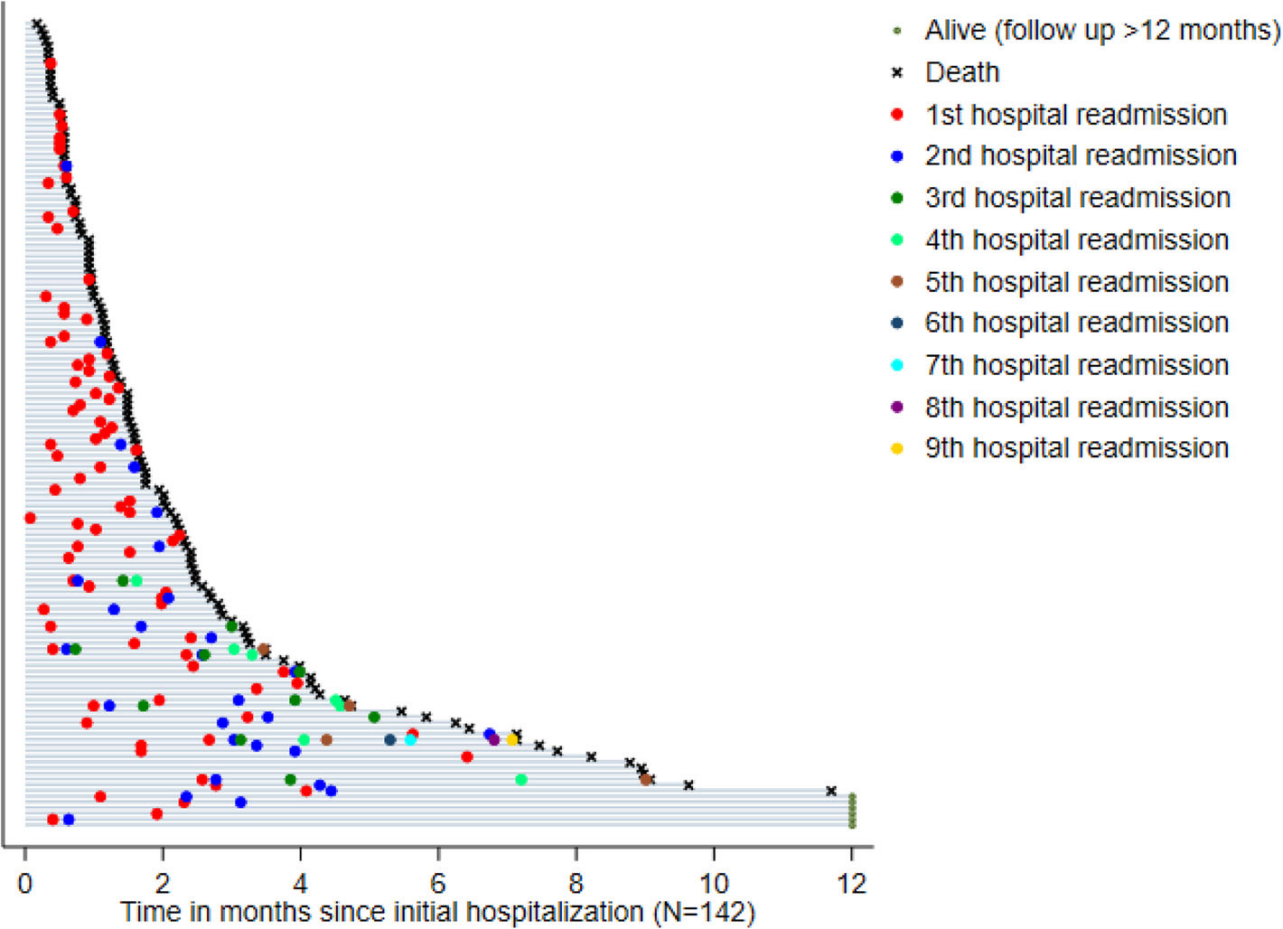
Swimmer plot representing individual trajectories and successive UHAs from initial hospital discharge to death or last follow-up Each horizontal bar represents an individual trajectory from initial hospital discharge to death or last follow-up. The dots symbolize the repeated UHAs. We have sorted the observations by the overall survival duration. Only the first 12 months of follow-up are represented on this figure; further follow-up and UHAs occurring beyond 12 months are not represented for the 6 patients alive for more than 12 months (observations censored at 12 months)

The occurrence of multiple UHAs within 12 months of the first hospital discharge is illustrated in Fig. 1. Fifty-four patients had one UHA, and 28 had two or more UHAs. After the initial discharge, the estimated mean number of UHAs was 1 on day 34 after initial hospital discharge, 2 on day 93, 3 on day 124, and 4 on day 186.

Considering the multiple home hospitalizations if any, the median total duration of the home hospitalization was 18.5 days (range, 1-252), and the mean duration was 38.5 days (standard deviation = 52.3).

### Place of death and overall survival duration

At the cut-off date, 138 of the 142 patients had died. The place of death was patient’s home (*n* = 71, 52.2 %), hospital (*n* = 65, 47.8 %), and unknown location (missing data, *n* = 2). The median overall survival time following the initial hospital discharge was 28 days (95 %-CI: 22–42; range: 1–1,092 days).

In univariate logistic regression analysis, we identified: Karnofsky index at the time of initial hospital discharge > 30 % (OR = 2.02; 95 %-CI, 1.01–4.03; *p* = 0.046), hospitalization in the patient’s home (OR = 3.86 ; 95 %-CI, 1.02–14.6 ; *p* = 0.046), living alone (OR = 2.59; 95 %-CI, 1.07–6.29; *p* = 0.035), and the presence of one or more children at home (OR = 3.55; 95 %-CI, 1.29–9.76; *p* = 0.014) as statistically associated with death in hospital. In the multivariate analysis controlling for the other potential confounding factors, the two significant factors for death in hospital were living alone (adjusted OR = 2.70; 95 %-CI, 1.01–7.2; *p* = 0.047) and the presence of one or more children at home (adjusted OR = 3.86; 95 %-CI, 1.14–13.1; *p* = 0.003) (Table 4).

**Table 4:** – Factors associated with death in hospital (univariate and multivariate analyses)

Parameters	Categories	n	Crude OR (95 %CI)	p	Adjusted OR^1^ (95 %-CI)	p
Sex	Women Men	83 53	1 0.96 (0.48–1.91)	0.91		
Age (/10 years)		136	0.82 (0.63–1.07)	0.15	0.89 (0.63–1.26)	0.51
Karnofsky index	≤ 30 % > 30 %	79 57	1 2.02 (1.01–4.03)	0.046	1 2.04 (0.92–4.56)	0.08
Primary tumor site	Breast Head and neck Digestive Gynecological Lung Urological Sarcoma / other	40 26 20 15 14 11 8	1 0.78 (0.29–2.09) 0.74 (0.25–2.17) 0.79 (0.24–2.60) 0.90 (0.27–3.06) 0.75 (0.19–2.88) 0.54 (0.11–2.58)	1.0		
Home	Patient home Other	117 14	3.86 (1.02–14.6) 1	0.046	2.66 (0.64–11.1) 1	0.18
Marital status	Single Other	40 89	1 0.66 (0.31–1.41)	0.29		
Patient living alone	No Yes	108 27	1 2.59 (1.07–6.29)	0.035	1 2.70 (1.01–7.2)	0.047
Caregiver at home	No Yes	15 100	1 1.01 (0.34-3.00)	0.98		
One or more children at home	No Yes	112 22	1 3.55 (1.29–9.76)	0.014	1 3.86 (1.14–13.1)	0.003
Initial discharge from	Medical oncology dept. Palliative care unit	82 54	1 1.03 (0.51–2.03)	0.95		
Length of hospital stay before discharge	Per 10-day increment	136	1.20 (0.92–1.57)	0.17	1.19 (0.86–1.64)	0.30
Psycho-oncology consultation	No Yes	99 36	1 2.04 (0.94–4.46)	0.07	1 1.80 (0.75–4.32)	0.19
Written advance directives	No Yes	129 7	1 1.48 (0.32–6.90)	0.61		
Preemptive prescription of end-life sedation	No Yes	112 24	1 0.60 (0.24–1.48)	0.27		

1) Multivariate regression model with 133 observations including the following variables: age, Karnofsky index, home, patient living alone and one or more children at home

## Discussion

### Main findings

Of the 142 study participants, more than 40 % of cancer patients receiving end of life home hospitalization were not readmitted to hospital. Importantly, a high proportion of the UHAs occurred soon after discharge to home. Most UHAs were related to physical symptoms and were initiated by the patient’s family physician. A post-discharge palliative care consultation was associated with a significantly lower incidence of UHAs. More than half of the deaths (52.2 %) occurred at home. In a multivariate analysis, living alone and having one or more children at home were associated with death in hospital.

### What this study adds

Many studies have shown that home care by a palliative care team is associated with less frequent hospital admissions in the last few months of life, fewer emergency room visits, and a shorter length of stay [18–24].

Furthermore, several studies have shown that quality of life is greater among patients who die at home than among patients who die in hospital, and family members much prefer the patient to die at home, even if of course cultural or socio-economic factors can influence this choice [25–29].

However, it is unreasonable to think that all patients can receive palliative care at home. Financial precariousness [5], carer exhaustion [30], and carer health problems [31] are reportedly factors for readmission to hospital but were not explored in the present study. Furthermore, UHAs with a medical indication can also be influenced by the psychosocial context at the patient’s place of residence [32].

Age and sex were not found to be associated with UHAs. Evidence from the literature on these factors is contradictory. With regard to sex, for example, Whitney et al. [33] and Jordhoy et al. [34] found that women were more likely than men to be readmitted to hospital. In contrast, Chang et al. [35] and Seow et al. [36] found that male sex was a factor associated with readmission to hospital. According to Riolfi and Chang, older age was associated with a lower risk of readmission [23, 35]. Seow et al. reported that patients aged 80 and over were hospitalized less frequently during the last two weeks of life [36].

Our descriptive data showed that the family physician is the primary initiator of UHAs. More than half the UHAs were triggered by an acute intercurrent event: this confirms data from a French nationwide study published in 2013 [14]. The particular logistic requirements of end-of-life home hospitalization may sometimes be incompatible with out-patient treatment, as emphasized in a 2017 report from the French government [31]. This might prompt family physicians to refer the person for inpatient treatment. The difficulties faced by the family physician in a context of end-of-life home hospitalization have been described in the literature: many family physicians see palliative care as a negative experience [37–39]. The family physician is in the front line and - on average - only deals with 1 to 3 home-based end-of-life situations per year [40].

Follow-up by a palliative care specialist might diminish the risk of a UHA. Van der Plas et al. reported that patients were more frequently readmitted to hospital when palliative care was coordinated by a family physician, compared with coordination by a specialist palliative care nurse [10]. Other studies have shown that a palliative care consultation might decrease the risk of readmission to hospital at 30 days [41, 42].

Nevertheless, the data concerning the effectiveness of follow-up consultations are divergent. Di Martino et al.’s meta-analysis did not find any convincing data on the putative influence of a home visit by a specialized palliative care professional on the likelihood of readmission to the emergency room [43]. Verhaegh et al.’s 2014 meta-analysis of patients suffering from chronic diseases found that the organization of face-to-face or phone consultations with a physician did not decrease the number of readmissions to hospital within 30 days of discharge to home. However, these consultations were associated with a lower number of readmissions to hospital for periods beyond the first 30 days [44].

Moreover, the fact that patients discharged from palliative care unit have a higher probability of being readmitted than those discharged from department of medical oncology can be explained by the specific mission of palliative care units, which is precisely to take in patients with most complex and difficult conditions.

With regard to the place of death, several variables selected in our study have been described in the literature as associated with an increased probability of dying at home: poor functional status [45–47], the presence of a caregiver [12, 45, 46, 48–52], and a wish to die at home expressed by the patient [45, 48–50] or by relatives [53]. In the present study, the last two criteria were initially selected but could finally not be studied due to a high proportion of missing data.

We have not observed any significant association between the age or sex and place of death. The literature data on the influence of age and sex on the place of death are heterogeneous. Several studies have not identified a significant link with these variables [24, 46, 48, 50, 52, 54], whereas some studies concluded that the probability of death at home was higher among elderly subjects [22, 36, 55, 56] or that women were more likely to die at home [52, 57].

Gomes et al.’s meta-analysis found that literature data on the factors influencing the place of death for patients with late-stage cancer are discordant. The researchers identified sources of bias due to (i) the retrospective design of most of the studies, (ii) changes in disease progression as a function of the cancer stage, and (iii) logistic problems that constrained the place of death [53].

In our study, only two factors were found to be associated with death at the hospital: living alone and the presence of one or more children at home. If Gomes has already reported that living with relatives was strongly associated with home death [53], the second factor has not previously mentioned in the literature to the best of our knowledge.. These results nevertheless confirm the daily experience of palliative care teams: the isolation of the patient or the presence of children at home, especially young children, represent hurdles to the anticipation of a possible death at home.

Finally, it should be emphasized that 42.2 % of the patients in home hospitalization were not readmitted, and 52.2 % died at home. The latter figure in particular, is higher than the national rate of death at home of around 25 %, all causes combined [58]. These figures therefore plead in favor of the advantage of home care hospitalizations in end-of-life situations.

### Limitations and strengths

The present study’s main limitation is inherent to its retrospective design. Data on some variables such as the presence of a caregiver at home were frequently missing; some others such as the patient’s wish to die at home could not be studied because they were not available in most patient files. The relatively small number of patients also limits the power of the analyses and our ability to introduce interaction terms in the multivariate regression models. This single-center study was performed in the specific context of palliative care in northern France - an area with high levels of home hospitalization and a well-organized palliative care network. Having included only the patients followed by the home hospitalization provider Santelys was both a strength and a weakness: a strength because it allowed a homogeneous collection of data, a weakness because it limits the external validity of the study. However Santelys is the main hospitalization provider of our center and all eligible patients followed by this home hospitalization provider were included in the study, reducing the risk of selection bias. We acknowledge that it may be difficult to extrapolate our findings to other areas, home hospitalization providers and healthcare systems.

One of the study’s strengths was its precise definition of a UHA with appropriate statistical methods for estimation, as recommended by Fischer et al.’s literature review [59]. The in-depth analysis of causes of UHA is also original.

### Perspectives

The IGAS (Inspection Générale des Affaires Sociales - *General Inspectorate of Social Affairs*) 2017 report [31] described three main causes of UHAs linked specifically to palliative care: (i) poor organization (notably a break in the chain of care), (ii) unreasonable therapeutic obstinacy, and (iii) poor anticipation of situations that are “*treatable at home*” but “*worrying for the patient*” (and probably also for the patient’s relatives). This lack of anticipation was confirmed in the IGAS 2018 report [5]; one consequence is the high proportion of UHAs via the emergency room (affecting 38.3 % of the patients in our study) [40].

Our present data showed that most of the patients with home palliative care were discharged from oncology departments. Discharge to home in these complex situations should be underpinned by a mobile palliative care team, since this may notably improve the degree of coordination with the family physician. The HAS recommends a visit by a family physician after any hospital stay of more than 24 hours [60]. In fact, less than half of the patients discharged from an oncology department had had a consultation with the mobile palliative care team.

Issuing advance directives can also help to plan end-of-life care and limit unreasonable therapeutic obstinacy. However, our present data did not highlight a significant link; only 5 % of the patients had drafted advance directives. This proportion is similar to that quoted by Pennec et al. (2.5 %) in a survey of 4723 end-of-life patients in France in 2010 [61]. Advance directives are infrequently used, even in a palliative context. In fact, tackling advance directives is stressful for both the patient (who has to confront his/her chronic and ultimately fatal disease) and the physician (who is unsure of how the disease will progress) [62].

## Conclusions

Our results showed that more than 40 % of cancer patients receiving palliative care at home, coordinated by the family physician and the homecare provider, were not readmitted to hospital. This proportion testifies to the effectiveness this type of home hospitalization, and emphasizes the importance of considering ways of improving home care procedures in a palliative setting.

Over half of the UHAs were due to an acute intercurrent event. Our results suggest that more effort should be focused on anticipating these events at home – primarily via better upstream coordination between hospital physicians and family physicians. Regular patient follow-up at the patient’s home by a family physician is essential, and the follow-up by an inpatient palliative care team might facilitate the anticipation and management of these acute intercurrent events. Prospective studies of the benefits of early home-based follow-up by a palliative care team are now required.

## Data Availability

The datasets used and/or analysed during the current study are available from the corresponding author on reasonable request.

## Abbreviations

IGAS: Inspection Générale des Affaires Sociales - *General Inspectorate of Social Affairs*.
PCA: Patient-Controlled Analgesia.
UHAs: Unplanned Hospital Admissions.
